# Mineral and bone disorder in hemodialysis patients in the Tibetan Plateau: a multicenter cross-sectional study

**DOI:** 10.1080/0886022X.2019.1635892

**Published:** 2019-07-04

**Authors:** Zong-Hui Dang, Chen Tang, Guo-Liang Li, Ciren Luobu, De Qing, Zhen-Hua Ma, Jing-Feng Qu, lamu Suolang, Li-Jun Liu

**Affiliations:** a Renal Division, The People’s Hospital of Tibet Autonomous Region, Lhasa, China;; b Renal Division, Peking University First Hospital, Beijing, China;; c Institute of Nephrology, Peking University, Beijing, China;; d Key Laboratory of Renal Disease, Ministry of Health of China, Beijing, China;; e Renal Division, The People's Hospital, Shannan, China;; f Renal Division, The People's Hospital, Shigatse, China;; g Renal Division, Second People's Hospital of Tibet Autonomous Region, Lhasa, China;; h Renal Division, The People's Hospital, Linzhi, China

**Keywords:** End stage renal disease, hemodialysis, MBD, high altitude, Tibet

## Abstract

**Background:** Mineral and bone disorder (MBD) in hemodialysis patients is associated with increased morbidity and mortality. Studies on the MBD status of hemodialysis patients at high altitudes are extremely limited.

**Methods:** A total of 146 hemodialysis patients from 5 local hospitals across all districts with hemodialysis centers in the Tibetan Plateau were enrolled in this cross-sectional study. Parameters related to MBD, including serum phosphorus (P), calcium (Ca), and intact parathyroid hormone (iPTH) levels, were measured. The achievement of MBD goals was compared with the achievement in the Dialysis Outcomes and Practice Study (DOPPS) 3, DOPPS 4 and a multicenter study of MBD in China. Factors associated with hyperphosphatemia were examined.

**Results:** Altogether, 146 hemodialysis patients were recruited from the Tibetan Plateau. According to the K/DIGO guidelines, there were low achievement rates for serum Ca (40.4%), P (29.7%), and iPTH (47.1%). As for the (KDOQI) guidelines, the rates of achievement of defined targets were 38.4%, 33.7% and 16.4% for serum Ca, P and iPTH, respectively. The percentages of patients reaching the KDOQI targets for corrected Ca, P, and iPTH were significantly lower for Tibetan patients than the percentages found in DOPPS 3 (38.4% vs. 50.4%, 33.7% vs. 49.8%, and 16.4% vs. 31.4%, respectively, all *p* < .001) and DOPPS 4 (38.4% vs. 56.0%, 33.7% vs. 54.5%, and 16.4% vs. 35.3%, respectively, all *p* < .001). The percentage of patients reaching the KDOQI targets for iPTH was significantly lower in Tibet than in the plain areas of China (16.4% vs. 26.5%, *p* < .001). The proportion of patients with hypocalcemia was higher in Tibet than in the plain areas (44.5% vs. 19.4%, *p* < .001). The percentage of local patients with optimal P was significantly higher for patients with an activated vitamin D prescription than for patients without an activated vitamin D prescription (45.3% vs. 19.3%, *p* < .001). Age and the activated vitamin D prescription were independently associated with hyperphosphatemia.

**Conclusion:** The MBD status of hemodialysis patients in Tibet is far from the ideal level. High altitude is one of the possible causes of the differences found, but not the principal one. It is necessary for medical staff in Tibet to improve the detection and treatment of MBD.

## Introduction

Mineral and bone disorder (MBD) is one of the important complications caused by chronic kidney disease (CKD), especially in hemodialysis patients, and MBD can cause morbidity and decrease quality of life in patients with CKD [1]. Patients with MBD have abnormalities related to mineral metabolism (e.g., nonoptimal serum levels of phosphorus, calcium, parathyroid hormone (PTH); altered bone structure and composition; and extraskeletal calcification) [2]. Among them, hyperphosphatemia is a key contributor to MBD [3].

Since 2007, the Dialysis Outcomes and Practice Pattern Study (DOPPS) program has reported the state of MBD in hemodialysis patients in several developed countries, such as the UK, USA, Japan [4]. This study found associations between mineral metabolism indicators and mortality and identified categories with the lowest mortality risk [4]. In recent years, there have been more reports about MBD in China, and a multicenter cross-sectional survey has also been carried out [5]. Moreover, guidelines from the Impact of the Kidney Disease Outcomes Quality Initiative (KDOQI) and Kidney Disease: Improving Global Outcomes (KDIGO) for CKD-MBD diagnosis, assessment, prevention, and treatment have been released and updated [6]. However, these studies were mostly focused on people in the plain areas, and studies regarding the status of MBD in hemodialysis patients at high altitudes are extremely limited. Tibet is a distinct area with a specific culture and lifestyle, and in this community, the residents spend their whole lives at high altitude [7]. Hence, we evaluated MBD and reported the MBD status of hemodialysis patients in the Tibetan Plateau to reflect the level of MBD control in hemodialysis patients at high altitudes. In this way, we can further improve the management of end-stage renal disease (ESRD) at high altitudes and improve their outcomes.

## Materials and methods

### Study population

**Figure 1.**
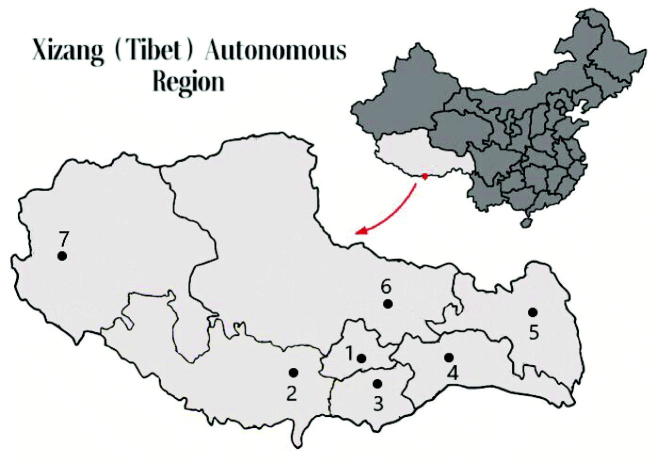
Hemodialysis centers in the Xizang (Tibet) Autonomous Region. (1) The People’s Hospital of Tibet Autonomous region and Second people's Hospital of Tibet Autonomous region (Altitude 3650 m); (2) Shigatse People’s Hospital (Altitude 4000 m); (3) Shan Nan People’s Hospital (Altitude 3700 m); (4) Lin Zhi District People’s Hospital (Altitude 3100 m); (5, 6 and 7) Districts without hemodialysis centers.

There were 7 hospitals across the Xizang (Tibet) region which can provide hemodialysis treatment. From them, patients who received regular hemodialysis for more than 3 months were enrolled in this study. Until May 2018, the medical records of 146 HD patients were reviewed and the demographic data and CKD-MBD laboratory marker levels were compiled from 5 hospitals of them. (One hospital, with 30 HD patients refused to take part in this study, and another hospital with HD patients less than 10 was excluded in the study since the sample size) A map marked with local hemodialysis centers is provided in Figure 1. The exclusion criteria included acute renal failure, chronic renal failure that did not require long-term regular dialysis, malignant tumors, active infections, severe liver failure or liver dysfunction (19 HD patients were excluded). In this study, all Helsinki regulations were followed, and all participants gave written informed consent prior to data collection.

### Data collection

Information on the general demographic characteristics of patients, the primary causes of ESRD, complications, the basic characteristics of dialysis (access type, filters, duration of dialysis, HD platforms, frequency), and the use and type of MBD medication were collected. Blood samples were drawn after an overnight fast of at least 8 h before dialysis. Parameters related to MBD, including hemoglobin (Hb), serum albumin (ALB), alkaline phosphatase (Alp), serum phosphorus (P), calcium (Ca) and intact parathyroid hormone (iPTH) concentrations, were measured at the laboratory of each local hospital. Serum phosphorus and serum calcium were measured by a spectrophotometry assay. iPTH was measured by immunoradiometric or immunochemiluminometric assays. Corrected calcium was calculated as total calcium (mg/dL) + 0.2×(4 − serum albumin (g/dL)).

### Statistical analyses

Normally distributed data are presented as the mean ± standard deviation (SD), and nonnormally distributed data are presented as the median with the interquartile range (Q25, Q75). Categorical data are summarized as counts and percentages.

According to the KDOQI guideline [8], patients with corrected Ca from 2.10 to 2.37 mmol/L (8.4 to 9.5 mg/dL), serum phosphorus from 1.13 to 1.78 mmol/L (3.5 to 5.5 mg/dL) and iPTH from 150 to 300 ng/L (150 to 300 pg/mL) were considered to be within the optimal range.

The K/DIGO initiative [9] recommend to maintain within the normal range for serum.

Calcium from 2.13 to 2.50 mmol/L (8.5 to 10.0 mg/dL) and phosphorous from 0.81 to 1.45 mmol/L (2.5 to 4.5 mg/dL) and within two to nine times the upper limit of the normal range for serum PTH (150 to 600 ng/L).

The percentages of participants within the optimal range and outside the optimal range (above or below) were compared with the results of DOPPS 3 (2007) and DOPPS 4 (2010) [4] and a multicenter study of MBD in Chinese dialysis patients [5].

The variables of groups were compared using independent-samples t-test (for normally distributed, continuous variables), Wilcoxon signed-rank tests (for nonnormally distributed, continuous variables) or χ^2^ tests (for nominal variables) as appropriate. Factors associated with hyperphosphatemia were analyzed using logistic regression models. Factors in the models included gender, age, duration of dialysis, hemoglobin level, calcium intake, vitamin D level, and phosphorus binder use.

All analyses were performed using the SPSS Statistics, version 22.0(SPSS Inc., Chicago, IL, USA). A *p* value less than 0.05 was considered statistically significant.

## Results

Altogether, 146 hemodialysis patients from the Tibetan Plateau were recruited, including 4 members of Han Chinese ethnicity and 142 Tibetans. The median age of these patients was 51 (36.5, 59), and 71.2% of them were male. The median vintage (month) of hemodialysis was 24 (13, 46) months. Most of them had different degrees of anemia. Among all the patients, 69 (47.3%) had chronic glomerulonephritis, 29 (19.9%) had diabetic nephropathy, 12 (8%) had hypertensive renal disease, 2 (1%) had interstitial nephritis, 1 (1%) had kidney obstruction, and the others had an unknown cause of ESRD (23%). Seventy-five of 146 patients were treated with activated vitamin D (calcitriol) regularly, and 75 out of 146 patients were treated with phosphorus binders. Regarding the duration and frequency of hemodialysis, the duration of each dialysis was 4 h for all patients, 95 (65.1%) patients received hemodialysis three times a week, 35 (24%) patients received hemodialysis twice a week, and 16 (10.9%) patients received hemodialysis only once a week. Moreover, 34 of them had different kind of symptoms of bone disease (Table 1).

**Table 1.** Characteristics of all the participants.

All participants (*n* = 146, %)			
Age (years)	51 (36.5, 59)	P binder prescription (%)	75 (51.4%)
Gender		Only Calcium-based	48 (32.9%)
Male (%)	104 (71.2%)	Only Sevelamer	12 (8.2%)
Female (%)	42 (28.8%)	Calcium-based + Sevelamer	14 (9.6%)
Hemodialysis Vintage (month)	24 (13, 46)	Other P binders	1 (0.7%)
Hb (g/L)	111.69 ± 25.29	Activated vitamin D use	75 (51.4%)
<90	29 (19.8%)	Calcium intake (g/day)	
90–100	20 (13.7%)	0	56 (38.4%)
100–110	19 (13.0%)	0.5	63 (43.2%)
110–120	23 (15.8%)	0.9	10 (6.8%)
120–130	20 (13.7%)	1	1 (0.7%)
130–140	14 (9.6%)	1.2	8 (5.5%)
>140	21 (14.3%)	1.5	8 (5.5%)
Etiology of renal failure		Cinacalcet use	16 (11.0%)
Chronic glomerulonephritis	69 (47.3%)	Symptoms of bone disease	34 (23.3%)
diabetic nephropathy	29 (19.9%)	Ostealgia	28 (19.2%)
hypertensive renal disease	12 (8%)	Skeletal deformity	1 (0.7%)
interstitial nephritis	2 (1%)	Others	5 (3.4%)
kidney obstruction,	1 (1%)	Dialysis frequency	
unknown cause	33 (23%)	Three times a week	95 (65.1%)
Dialysis access type		Twice a week	35 (24%)
AVF (arteriovenous fistula)	35 (24.0%)	Once a week	16 (10.9%)
TCC (tunneled cuffed catheter)	111 (76.0%)	Duration of dialysis	Four hours each time

In addition, the Filters available in Tibet included Polyflux 14 L, 170 H and Revaclear 400 from Gambro; HD-150 and HD-180 from OCI. The Platforms included AK96 from Gambro, 4008 s from Fresenius, Dialog + from Braun.

**Figure 2.**
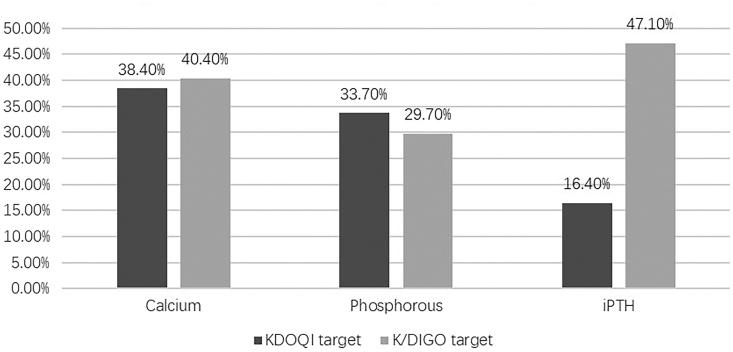
Percentage of patients within KDOQI and K/DIGO target for serum calcium, phosphorous and iPTH.

According to the K/DIGO guidelines, there were low achievement rates for corrected calcium (40.4%), serum phosphorus (29.7%), and iPTH (47.1%). As for the KDOQI guidelines, only 38.4%, 33.7% and 16.4% of MBD patients in the Tibet Plateau met the defined targets for corrected calcium, serum phosphorus, and iPTH levels, which were significantly lower than the percentages found in DOPPS 3 (50.4%, 49.8%, 31.4%, respectively, all *p* < .001) and DOPPS 4 (56.0%, 54.5%, 35.3%, respectively, all *p* < .001) (Table 2, Figure 2).

**Table 2.** Measurement of biochemical parameters.

All participants (*n* = 146)			
Corrected Calcium (mmol/L)	2.15 ± 0.26		
Phosphorus (mmol/L)	1.79 ± 0.67		
iPTH (ng/L)	511.45 (267.35, 889.87)		
KDOQI initiative		K/DIGO initiative	
Corrected Calcium (*n*, %)			
<2.10 (*n,* %)	65 (44.5)	<2.13 (*n*, %)	74 (50.7)
2.10–2.37 (*n*, %)	56 (38.4)	2.13–2.50 (*n*, %)	59 (40.4)
>2.37 (*n*, %)	25 (17.1)	>2.50 (*n*, %)	13 (8.9%)
Phosphorus (mmol/L)			
<1.13 (*n*, %)	27 (18.6)	<0.81 (*n*, %)	6 (4.1)
1.13–1.78 (*n*, %)	49 (33.7)	0.81–1.45 (*n*, %)	43 (29.7)
>1.78 (*n*, %)	69 (47.6)	>1.45 (*n*, %)	96 (66.2)
iPTH (ng/L)			
<150 (*n*, %)	17 (12.1)	<150 (*n*, %)	17 (12.1)
150–300 (*n*, %)	23 (16.4)	150–600 (*n*, %)	66 (47.1)
>300 (*n*, %)	100 (71.4)	>600 (*n*, %)	57 (40.7)

The percentage of patients with optimal iPTH levels was significantly lower in the Tibetan Plateau than in the plain areas of China (16.4% vs. 26.5%, *p* < .001). The proportion of patients with hypocalcemia was higher in Tibet than in the plain areas (45.9% vs. 19.4%, *p* < .001). Additionally, the proportion of patients with hypophosphatemia in Tibet was evidently higher than the proportion found in DOPPS 3 (18.5% vs. 11.0%, *p* < .001), DOPPS 4 (18.5% vs. 11.3%, *p* < .001) and other plain areas of China (18.5% vs. 5.0%, *p* < .001) (Table 3).

**Table 3.** Comparison of mineral metabolism laboratory parameters between patients at high altitude and patients from DOPPS 3, DOPPS 4 and other plain areas of China.

	Sample (*n*, %)	DOPPS 3 (*n*, %)	*p*	DOPPS 4 (*n*, %)	*p*	Plain areas (*n*, %)	*p*
Corrected Calcium (mmol/L)							
<2.10	65 (44.5)	791 (12.5)	<.001	904 (12.4)	<.001	324 (19.4)	<.001
2.10–2.37	56 (38.4)	3195 (50.4)		4080 (56.0)		647 (38.6)	
>2.37	25 (17.1)	2355 (37.1)		2300 (31.6)		703 (42.0)	
Phosphorus (mmol/L)							
<1.13	27 (18.5)	765 (11.0)	<.001	895 (11.3)	<.001	86 (5.0)	<.001
1.13–1.78	49 (33.72)	3476 (49.8)		4377 (54.5)		642 (37.6)	
>1.78	69 (47.3)	2738 (39.2)		2722 (34.2)		981 (57.4)	
iPTH (ng/L)							
<150	17 (12.1)	2073 (36.8)	<.001	2325 (32.5)	<.001	473 (29.0)	<.001
150–300	23 (16.4)	1767 (31.4)		2299 (32.1)		431 (26.5)	
>300	100 (71.4)	1787 (31.8)		2527 (35.3)		724 (44.5)	

DOPPS 3 (2007) refers to the study of the prognosis and practice of dialysis in 2007, with data from Australia, Belgium, Canada, Germany, Italy, Japan, Spain, Sweden, UK, and the United States; DOPPS 4 (2012) added French data to the above regions.

 

The percentage of patients with optimal serum phosphorus levels was significantly higher for patients with an activated vitamin D prescription than those without an activated vitamin D prescription (45.3% vs. 19.3%, *p* < .001). The percentage of patients with higher iPTH levels was lower in the group with a vitamin D prescription than in the group without an activated vitamin D prescription (64.8% vs. 83.9%, *p* = .032). However, there was no significant difference in the percentage of patients with optimal Ca (42.7% vs. 33.3%, *p* = .211) between the two groups (Table 4).

**Table 4.** Comparison between the patients with a vitamin D prescription and the patients without a vitamin D prescription.

	VD prescription (*n* = 75)	No VD prescription (*n* = 57)	*p* value*
Age (years)	46.1 ± 14.9	53 (43, 60.5)	.714
Male (%)	69	78.9	.162
Duration of Dialysis	30.6 ± 24.9	33.1 ± 24.4	.003
Hb (g/L)	106.80 ± 24.88	115.74 ± 26.03	.047
Hemodialysis Vintage (month)	2.17 ± 0.28	2.13 ± 0.25	.364
Corrected Calcium (*n*, %)			
<2.10	28 (37.3)	30 (52.6)	.211
2.10–2.37	32 (42.7)	19 (33.3)	
>2.37	15 (20.0)	8 (14.0)	
Phosphorus (mmol/L)	1.61 ± 0.68	1.98 ± 0.62	.002
Phosphorus (*n*,%)			
<1.13	18 (24.0)	8 (14.0)	<.001
1.13–1.78	34 (45.3)	11 (19.3)	
>1.78	23 (30.7)	38 (66.7)	
P binder prescription (%)	53.3	61.4	.354
iPTH (ng/L)	447.5 (215.00, 804.00)	716.2 (308.9,1009.2)	<.001
iPTH (*n*,%)			
<150	11 (15.5)	2 (3.6)	.032
150–300	14 (19.7)	7 (12.5)	
>300	46 (64.8)	47 (83.9)	

In univariable regression, the use of activated vitamin D was associated with hyperphosphatemia (OR = 0.195, 95% CI, 0.082 to 0.465, *p ≤* .001). After adjusting for covariates by multivariable logistic regression, age (OR = 0.963, 95% CI: 0.929 to 0.998, *p* = .039) and prescription of vitamin D (OR = 0.140, 95% CI: 0.050 to 0.388, *p* ≤ .001) were both independently associated with hyperphosphatemia. Gender, age, hemoglobin level, calcium intake, and use of phosphorus binders were not associated with hyperphosphatemia (Table 5).

**Table 5.** Logistic regression analysis for hyperphosphatemia with different variables.

	Univariate			Multivariate		
Characteristics	OR	95% CI	*p*	OR	95% CI	*p*
Gender (male vs. female)	1.267	(0.537, 2.993)	.589	1.060	(0.387, 2.900)	.910
Age	0.983	(0.956, 1.011)	.231	0.963	(0.929, 0.998)	.039
Duration of dialysis	0.996	(0.981, 1.012)	.632	0.992	(0.974, 1.011)	.413
Hb	1.004	(0.988, 1.021)	.635	1.001	(0.981, 1.022)	.911
Ca Intake	0.374	(0.114, 1.231)	.106	0.809	(0.190, 3.446)	.775
Activated Vitamin D (use vs. no use)	0.195	(0.082, 0.465)	<.001	0.140	(0.050, 0.388)	<.001
Phosphorus binder (use vs. no use)	0.877	(0.386, 1.993)	.877	1.264	(0.448, 3.565)	.657

## Discussion

MBD, a common complication of CKD, is an important cause of morbidity, decreases quality of life, and leads to additional calcification that has been associated with increased cardiovascular mortality [2]. Many studies have reported progress and improvement in the treatment of MBD in dialysis patients in many countries [4]. However, studies regarding the status of MBD treatment in high-altitude areas are extremely rare. The Xizang (Tibet) Autonomous Region is located in the southwest area of the Qinghai-Tibet Plateau, where the average elevation is more than 4000 meters. Studying local patients in Tibet can reflect the status of MBD in hemodialysis patients at high altitudes. Under the influence of economy and transportation, there are only 7 hospitals in Tibet can offer hemodialysis service, 5 out of 7 were included in the study, hence, in this study, what we investigated from there can represent the current situation of local patients. Indeed, the current study has been the largest report of patients at high altitudes so far. Yet, from the results, we found low achievement rates for Ca, P, and iPTH according to the KDIQO target or the KDIGO target, and discrepancies with other studies, which suggested that the MBD status of hemodialysis patients in the Tibetan Plateau was not ideal.

Among them, hyperphosphatemia is an independent risk factor for both mortality and renal function decline in CKD patients [1,10]. The percentage of patients reaching the KDOQI target for serum phosphorus was significantly lower in Tibetan patients than in DOPPS 3 and DOPPS 4 patients. There are several possible explanations for the result. First, Tibetan people have a specific culture and lifestyle. Their daily diet is rich in phosphorous sources, including cattle, sheep, animal viscera, and animal fat and its byproducts (e.g., butter and milchig), which leads to a high daily intake of phosphorus. In Kong’s study, the issue of hyperphosphatemia was also prominent in other plain areas of China, partly because the local population consumed many phosphorous-rich foods [5]. To this point, dietary education is crucial to control blood phosphorus levels [11,12], reduce the incidence of secondary hyperparathyroidism, and reduce the use of calcium-based phosphorus binders [13]. Additionally, it is reasonable to consider phosphate sources in making dietary recommendations [14]. Second, Tibet is an area with relatively lower economic development and living standards than most other areas of the world. The prevalence of kidney disease is high in economically underdeveloped areas [15]. In addition, economic conditions also affect the treatment options for patients. Half of the patients (73/146) were not treated with phosphorus binders, 48 of them used calcium-based phosphorous only. Regarding hemodialysis frequency, only 65% of patients received hemodialysis treatment three times a week. Studies have shown that dialysis frequency is associated with adequate phosphorus clearance and that shortening the dialysis interval can reduce blood phosphorus levels more effectively [16]. Finally, environmental toxins may be involved in this problem. A recently published article shows a high methylmercury exposure level and environmental mercury burden in the Tibetan Plateau, which is generally recognized as the cleanest region in China [17]. The concentration of Hg is associated with decreased bone resorption and formation [18], which might affect bone mineralization and serum phosphorus levels.

Interestingly, hypophosphatemia is also notable in Tibet. The percentage of patients with hypophosphatemia in Tibet is evidently higher than in the DOPPS reports and other plain areas of China. Patients with hypophosphatemia, an important predictor of mortality, present with worse values of nutritional status markers and protein intake and higher levels of overhydration [19]. Therefore, we speculate that one of the causes of this might be related to the high percentage with weakness and the poor nutritional status of the local population. Longer follow-up studies are needed in Tibet to understand the trends for the future.

Logistic regression analysis also showed that the use of activated vitamin D as a protective factor was associated with hyperphosphatemia for local people, and patients taking vitamin D were less likely to develop hyperphosphatemia (Table 4). However, a meta-analysis concluded that vitamin D therapy cannot improve biochemical markers of MBD [20]. Generally, activated vitamin D can promote the absorption of calcium and phosphorus from the intestine but can also cause hypercalcemia and hyperphosphatemia in CKD [21]. In addition, current research suggests that vitamin D insufficiency and deficiency should be avoided in CKD and dialysis patients by using supplementation to prevent secondary hyperparathyroidism (SHPT) [22]. The findings of prolonged survival in some studies may be because patients prescribed vitamin D tended to be healthier overall than those who were not prescribed vitamin D, and therefore, the survival benefit may be due to the underlying health status of study participants rather than to the vitamin D treatment itself [4]. Hence, we assume this result may be due to similar reason: local patients were given vitamin D treatment on the premise that their blood phosphorus levels were controlled.

For the level of serum calcium control, the situation was as unsatisfactory as for the serum phosphorus control. The low calcium proportion of the population was high, although 51.3% of the patients in this study had an activated vitamin D prescription. Hypocalcemia is a typical feature of CKD. The development of renal failure in local patients can cause phosphate retention and influence calcium absorption. Moreover, the unique environment is another important cause. Tibet is a high-altitude area with a special climate, low air pressure, and low oxygen levels, where the residents live for their entire lifetime. Acclimatization at high altitude is often accompanied by decreased plasma calcium [23]. Moreover, local people in Tibet consume a large amount of brick tea, which contains fluorine [24]. Fluorine can form a compound with calcium, which obviously reduces the serum concentration of calcium. Hypocalcemia has a certain effect on human health and contributes to the development of SHPT, which is another independent predictor of cardiac and all-cause mortality in CKD patients [25]. Vitamin D and calcium supplementation are important for improving hypocalcemia. However, the recommendations should be individualized, mild and asymptomatic hypocalcemia could be tolerated to avoid inappropriate calcium loading in adults [14].

According to the KDOQI guidelines, only 16.4% of local patients achieved the target for iPTH, which was significantly lower than the proportion in other regions of China and DOPPS standards. Since the KDIGO guidelines were less restrictive, 47.1% of patients reached the goal for iPTH, but still, almost half of them still exceed that target. And about 23.3% of them already had the symptoms of bone disease (Table 1). It was thought that patients residing in higher altitudinal strata tended to have lower PTH levels, resulting from greater UV-B exposure at higher altitudes [26]. Low calcium and elevated phosphate concentrations of local patients undoubtedly contribute to the progress of hyperparathyroidism [27]. Surprisingly, we found serious renal anemia of local patients, nearly 1/3 of people had lower levels of hemoglobin than 100 g/L. A recent finding suggests that high PTH levels suppress the endogenous EPO synthesis and thereby contribute to renal anemia [28], which can possibly explain it. All in all, timely and appropriate treatment is crucial. Effective control of hyperphosphatemia would help to decrease iPTH levels in patients on maintenance hemodialysis [29] drugs like calcitriol, vitamin D analogs and other agents like cinacalcet can also control the level of iPTH [14], and among them, cinacalcet can even alleviate renal anemia [28], stabilize vascular calcification [30]. However, very few people took effective measures. For example, the percentage of patients using cinacalcet in this study was limited just because of economic reasons, let alone surgical parathyroidectomy.

The findings of this study must be considered in the context of the following limitations. First, the number of patients surveyed was limited, which might introduce bias. Second, some information related to the MBD was not available in our study, such as detailed dose of activated Vitamin D, 25-dihydroxy vitamin D levels, residual renal function, and details of diet habits. Third, all laboratory tests were not performed in the same lab. Fourth, there was a lack of follow-up of patients and observation of long-term events related to cardiovascular and cerebrovascular diseases.

## Conclusion

Due to the Plateau-specific factors, the patients in Tibet had a different status of CKD-MBD, local altitude, climate, and lifestyle all had an impact on the status to a certain extent, but proper treatment may be the most principal factors. On balance, the MBD status in hemodialysis patients in Tibet was far from satisfactory. It is necessary for local medical staff to improve the detection and treatment of CKD-MBD in hemodialysis patients and reduce the risk for vascular calcification and cardiovascular mortality.
