# Correction

**DOI:** 10.1080/0886022X.2021.1927335

**Published:** 2021-05-27

**Article title:** Mineral and bone disorder in hemodialysis patients in the Tibetan Plateau: a multicenter cross-sectional study

**Authors:** Zong-Hui Dang, Chen Tang, Guo-Liang Li, Ciren Luobu, De Qing, Zhen-Hua Ma, Jing-Feng Qu, lamu Suolang, and Li-Jun Liu

**Journal:** *Renal Failure*

**Bibliometrics:** Voume 41, Number 1, pages 636–643

**DOI:** 10.1080/0886022X.2019.1635892

When the above article was first published online, Taiwan province was missing from the map of China, which is Figure 1. This will be replaced with the updated figure and caption below:

These have now been corrected in the online version.

*Taylor & Francis respects its authors' decisions regarding the designations of territories in its published material. Taylor & Francis' policy is to take a neutral stance in relation to territorial disputes or jurisdictional claims in its published content, including in maps and institutional affiliations.*

**Figure 1.**
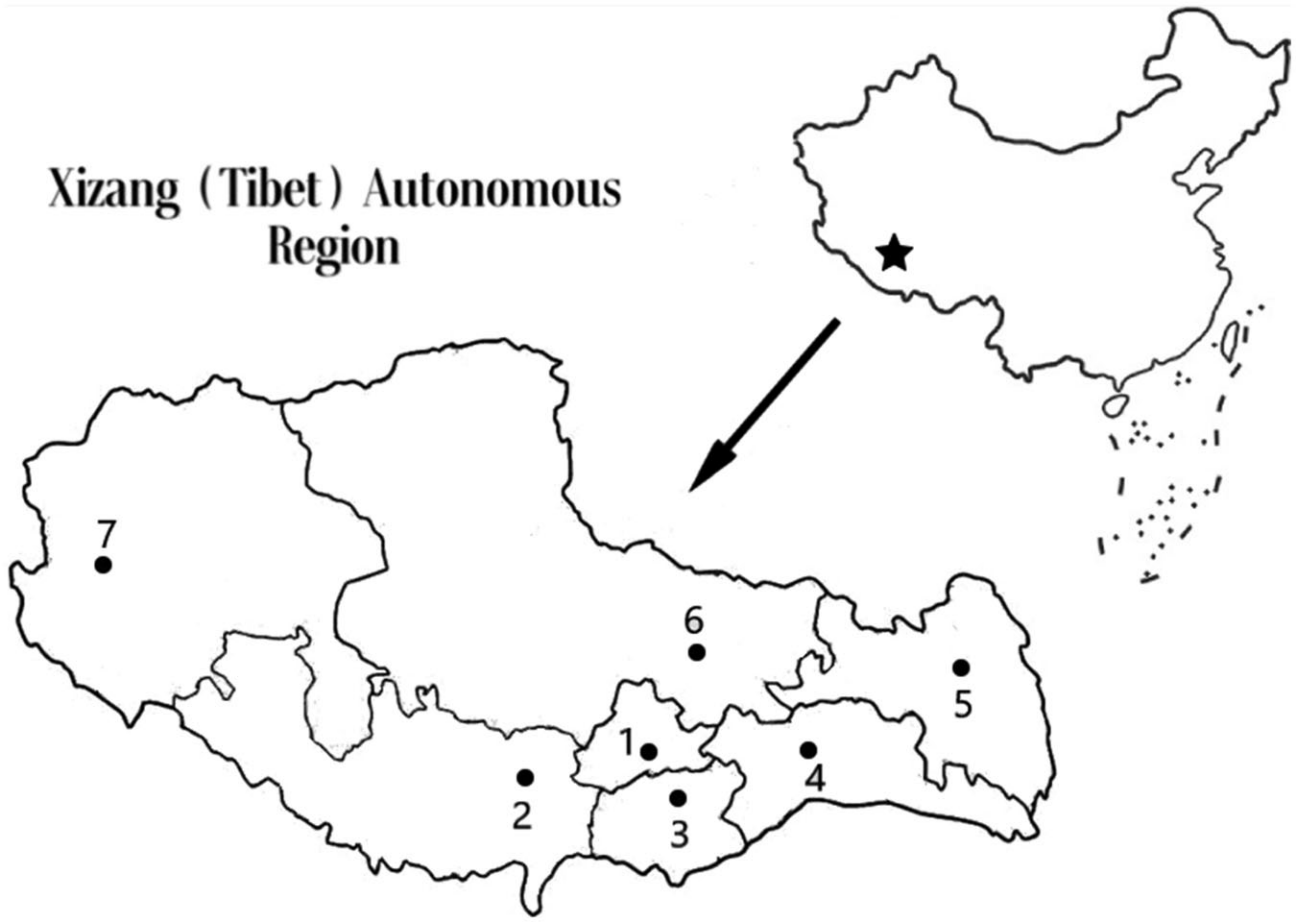
Hemodialysis centers in the Xizang (Tibet) Autonomous Region. (1) The People’s Hospital of Tibet Autonomous region & Second people's Hospital of Tibet Autonomous region (Altitude 3650 m); (2) Shigatse People’s Hospital (Altitude 4000 m); (3) Shan Nan People’s Hospital (Altitude 3700 m); (4) Lin Zhi District People’s Hospital (Altitude 3100 m); (5, 6 & 7) Districts without hemodialysis centers.

